# Selection and characterization of a broadly neutralizing class of HCV anti-E2 V_H_1-69 antibodies

**DOI:** 10.1371/journal.ppat.1012428

**Published:** 2025-03-28

**Authors:** Andreas Soerensen, Filip Popovic, Christina Holmboe Olesen, Blanca Lopez Mendez, Brian Lohse, Zhaochun Chen, Patrizia Farci, Robert H. Purcell, Harvey J. Alter, Lea Klingenberg Barfod, Jens Bukh, Jannick Prentoe

**Affiliations:** 1 Copenhagen Hepatitis C Program (CO-HEP), Department of Infectious Diseases, Hvidovre and Department of Immunology and Microbiology, Faculty of Health and Medical Sciences, Copenhagen University Hospital, University of Copenhagen, Copenhagen, Denmark; 2 Novo Nordisk Foundation Center for Protein Research, Faculty of Health and Medical Sciences, University of Copenhagen, Copenhagen, Denmark; 3 Department of Drug Design and Pharmacology, Faculty of Health and Medical Sciences, University of Copenhagen, Copenhagen, Denmark; 4 Laboratory of Infectious Diseases, National Institute of Allergy and Infectious Diseases, National Institutes of Health, Bethesda, Maryland, United States of America; 5 Department of Transfusion Medicine, Warren Grant Magnuson Clinical Center, National Institutes of Health, Bethesda, Maryland, United States of America; 6 Centre for Translational Medicine and Parasitology, Department of Immunology and Microbiology, Faculty of Health and Medical Sciences, University of Copenhagen, Copenhagen, Denmark; Heidelberg University, GERMANY

## Abstract

Identification and characterization of antibody epitope targets on the hepatitis C virus (HCV) envelope proteins remain crucial for developing an effective vaccine. Building on prior research defining E1/E2 antibody epitope clustering, we screened a phage display library derived from a chronic HCV patient against detergent-extracted full-length E1/E2 from both the patient’s acute-phase isolate (H77, genotype 1a) and a heterologous isolate (S52, genotype 3a). This approach yielded a panel of V_H_1-69 derived antibody fragments (Fabs) with similar characteristics. Interestingly, all members of the panel exhibited blocking activity against both antigenic region 2 and 3 (AR2 and AR3) in competition ELISAs, which contrasts with the behavior of most previously identified AR3-targeting antibodies. The V_H_1-69 derived binders had a high affinity for soluble E2 in both Fab and IgG formats, with dissociation constants in the low picomolar range. These Fab binders were broadly neutralizing against a panel of HCV cell culture viruses of genotype 1-6 with higher potency than the well-characterized reference Fab, AR3A. However, in the IgG format the antibodies had similar potency. These findings expand our understanding of potential targets for vaccine development by characterizing a panel of antibodies targeting an AR3 epitope also involving or occluding the back layer of E2. The broad neutralization and high affinity of these antibodies suggest a benefit to targeting both the back layer and the front layer of E2 in HCV vaccine designs to expand the repertoire of broadly neutralizing antibodies, thereby offering promise for the development of more effective preventive measures against this pervasive human pathogen.

## Author summary

We successfully isolated a panel of antibodies from a patient with chronic hepatitis C virus (HCV) infection. Surprisingly, these antibodies compete with antibodies targeting two distinct antigenic regions (AR2A and AR3A) on the HCV envelope simultaneously and exhibited extremely high affinity binding as well as broad neutralization capabilities on par with the most potent previously described AR3-like antibodies. This discovery expands our understanding of the immune response to HCV and provides valuable insights for future vaccine design. Our work contributes to ongoing efforts to develop more effective vaccines against HCV infection, with potential positive implications for global health. Further research into the mechanisms underlying the unique properties of these antibodies could pave the way for innovative vaccine strategies against HCV.

## Introduction

Chronic infection with hepatitis C virus (HCV) remains a global health concern, impacting over 50 million individuals worldwide [1]. Despite the availability of effective antiviral treatments, the persistent burden of HCV is exacerbated by limited access to diagnosis and care, particularly in resource-limited settings. Despite a salient need, more than 30 years since the characterization of the genome [2], a vaccine for HCV has not yet been developed. One key obstacle is the genetic diversity of the virus, comprising eight distinct genotypes (Gt) and over 90 subtypes [1,3,4]. This diversity is most prominent in the envelope glycoproteins E1 and E2 [5]. In addition to genetic variability, the virus can also be grouped into clusters based on antigenicity and neutralization susceptibility [6]. Therefore, to be effective, a protective vaccine against HCV would need to elicit broadly neutralizing antibodies (bNAbs), which are a specific group of antibodies that recognize and neutralize antigenically divergent viral strains by targeting conserved regions on the virus [7]. The envelope glycoproteins, E1 and E2, form a homodimer of E1/E2 heterodimers on the viral surface and represent the major target of B-cell recognition [8,9]. On the E1/E2 glycoproteins, the antigenic region 3 (AR3) and AR4 represent attractive targets for a protective HCV vaccine [10–12], as the bNAbs recognizing these regions have an exceptional breadth and potency, while also having a high barrier to resistance [13–15]. AR3 represents a cluster of discontinuous epitopes formed by the front layer and CD81 binding site of E2 [16]. Most AR3 bNAbs originate from the V_H_1-69 germline family, which is known for its role in mounting early, broad, and potent immune responses against HCV, as well as other viruses like influenza A virus (IAV) [17] and human immunodeficiency virus 1 (HIV-1) [18]. They are characterized by mainly interacting through the heavy chain with a hydrophobic tip on the complementarity determining region 2 (HCDR2) (Reviewed in ref. [19]).

For HCV, V_H_1-69 encoded bNAbs target AR3 by different angles of approach, while only needing few somatic hypermutations from the derived germline gene to neutralize the virus [12,20]. To develop a vaccine based on the AR3 epitope, it will likely be important to understand how to optimally expose this epitope so that the most broad and potent antibodies are consistently induced.

The chronic HCV patient, known as ‘patient H’ (pt. H), plays a pivotal role in this narrative of neutralizing antibodies against HCV. Pt. H.’s serum is known to contain high titers of bNAbs and the patient is also the original donor for the “H strain,” with the acute-phase H77 isolate emerging as a key model isolate in HCV research [21]. Although the patient never cleared the infection, immunoglobulin (Ig) preparations, such as the polyclonal IgG mix, H06, from pt. H, have demonstrated protective efficacy against Gt1-Gt7 in cell cultured HCV (HCVcc) [22], as well as against Gt1a, Gt4a, and Gt6a infection in a human liver chimeric mouse model [23]. Furthermore, the polyclonal IgG mix was also tested in a chimpanzee by infusing pre- and post-challenge with Gt1a, Gt4a, Gt5a, and Gt6a viruses. Here, suppression against the homologous Gt1a and protection against the heterologous Gt6a was observed, although with no effect against Gt4a and Gt5a [24]. These studies underscore the complexity of HCV infection and the challenges in targeting conserved epitopes, emphasizing the need for a more in-depth understanding of molecular and genetic determinants for broad antibody neutralization in HCV infection.

Here, we isolate and characterize a panel of closely related V_H_1-69-encoded bNAbs from a pt. H phage display library. The panel represents a potential new class of AR3-like antibodies that competes with both the front-layer binding antibody, AR3A, and back-layer binding antibody, AR2A, in competition ELISAs. This research indicates an important role of these highly potent antibodies in neutralizing HCV and further contributes to the growing literature on V_H_1-69 derived antibodies in viral immunology.

## Results

### Isolation of HCV-specific Fab-displaying phages

We constructed a phage display Fab library from bone marrow aspiration of pt. H and screened the library against detergent-extracted full-length E1/E2 from both a homologous (isolate H77, Gt1a [25]) and a heterologous (isolate S52, Gt3a [26]) source. A similar library had previously been constructed from pt. H and screened against a truncated soluble form of E2 from H77, which led to the selection and characterization of five HCV-specific antibodies, three from the V_H_1-69 germline and two from the V_H_4-59 germline [27]. Among these, only one V_H_1-69 Fab, designated HCV#4, exhibited broad neutralization, capable of neutralizing Gt1a, Gt1b, and Gt2a in HCV pseudo-particle assays. Given the documented virus control in pt. H, we hypothesized that additional bNAbs could be isolated from the patient [21].

**Fig 1:**
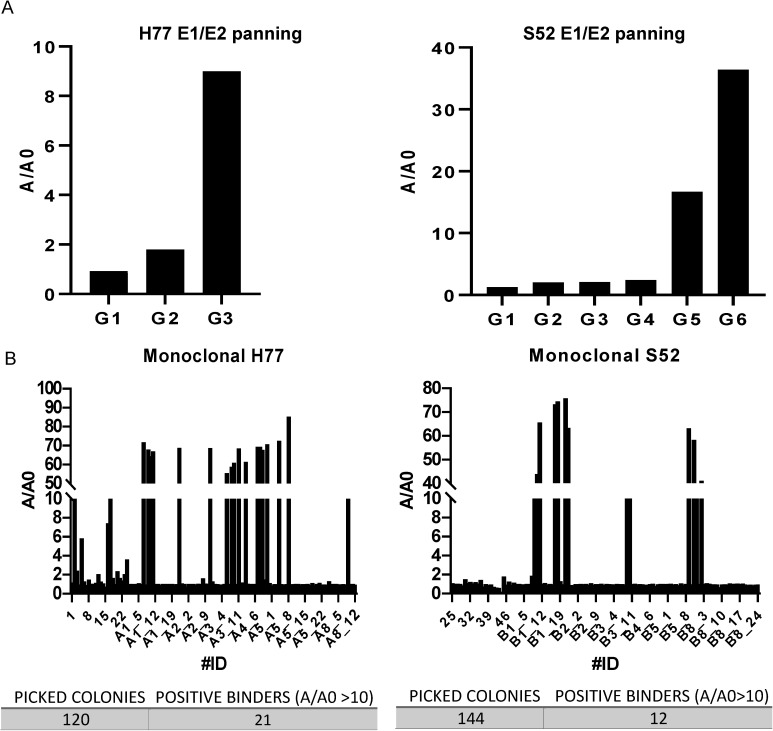
Screening of the phage library. A) Polyclonal and B) monoclonal ELISA of enriched libraries against detergent-extracted full-length E1/E2 of either the H77 or S52 isolate. ELISA measured at A450 and presented as absorbance measured to target (A) divided by absorbance measured to control (A0). Each generation of enriched phages are indicated as G1, G2, G3, etc. The increased signal from each round indicates that the libraries converge to positive binders that can then be isolated and validated by monoclonal ELISA. The monoclonal ELISAs of isolated colonies were set with a cut-off at 10 for A/A0 to limit potential false positives. The positive binders with a A/A0 value above 10 were then sequenced to retrieve the variable heavy chain (VH) and variable light chain (VL). We picked 120 colonies in total with 21 positive binders for the screening against H77 E1/E2 and 144 colonies in total with 12 positive binders for the screening against S52 E1/E2.

Following library expression and packaging into helper phages, we subjected it to three to six rounds of panning against either H77 or S52 E1/E2. The enrichment towards E1/E2 was assessed via polyclonal phage ELISA using the corresponding antigens (Fig 1).

**Fig 2:**
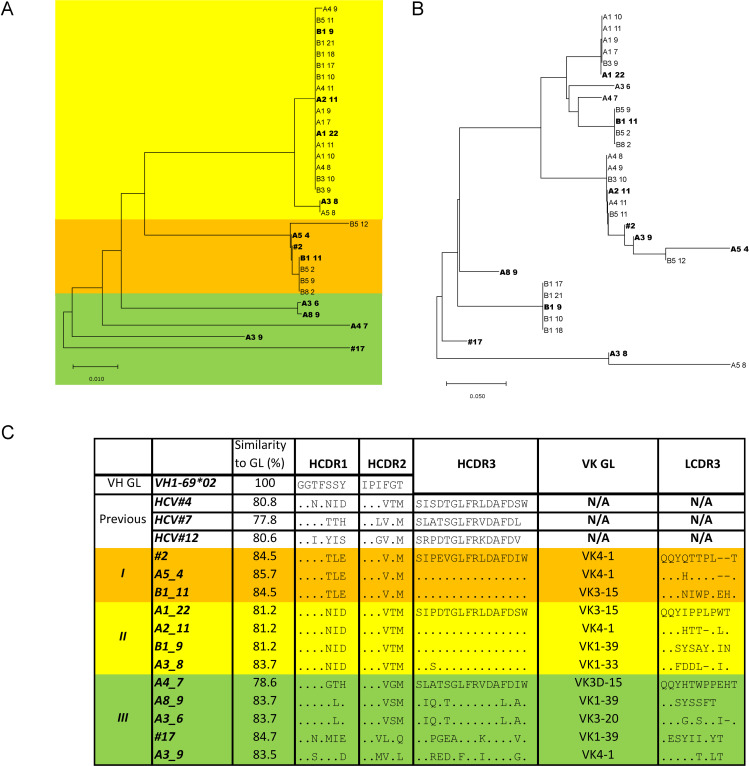
Identification of unique phage clones. A) Alignment and neighbor-joining phylogenetic tree of VH nucleotide sequences and B) of VL nucleotide sequences using MEGA v. 10.1.5. The unique sequences are marked with bold in A and B. Unique binders are determined as either having a unique VH or a unique VH/VL sequence combination. Colors represent each determined grouping of similarity. C) Alignment of CDR sequences, as defined by Chothia [28], of isolated binders and previously identified V_H_1-69 Fabs by Schofield et al. [27] to the closest related germline (GL) gene, V_H_1-69*02. Only the VH sequences for the previously identified V_H_1-69 were deposited. The similarity to GL were determined as the number of amino acids similar to the GL V sequence (Framework region 1 (FR1) to half of CDR3).

From the final round of panning, colonies were picked for monoclonal phage ELISA, identifying 33 E1/E2 binders. Sequencing of these 33 clones revealed 11 clones with unique sequences (i.e., unique variable heavy chain (VH) or variable light chain (VL) sequences, or a unique VH/VL pairing). Notably, all clones originated from the V_H_1-69 germline with minor VH variation, while specific VLs appeared to be favored, including V_κ_4-1, V_κ_3-20, V_κ_3-15, and V_κ_1-39 (Fig 2).

Clustering based on VH sequence similarity yielded three groups of binders, here named group I, II, and III. Group II VH sequences were most closely related to the previously isolated HCV#4 and for the group II clone, A3_8, only one amino acid was different in the HCDR1. We did not isolate any of the previously published VH sequences from pt. H described by Schofield et al. [27]. Of the 12 binders retrieved from the S52 panning, only B1_9 and B1_11 were unique, i.e., not found in the H77 panning rounds, although only by having unique VH/VL pairings (See S1 Data). The pairing of VH/VL is not necessarily the native pairing as found in the patient but is likely random due to the VH/VL reassortment involved when constructing the library.

### Binding kinetics and epitope specificity of Fabs

**Fig 3:**
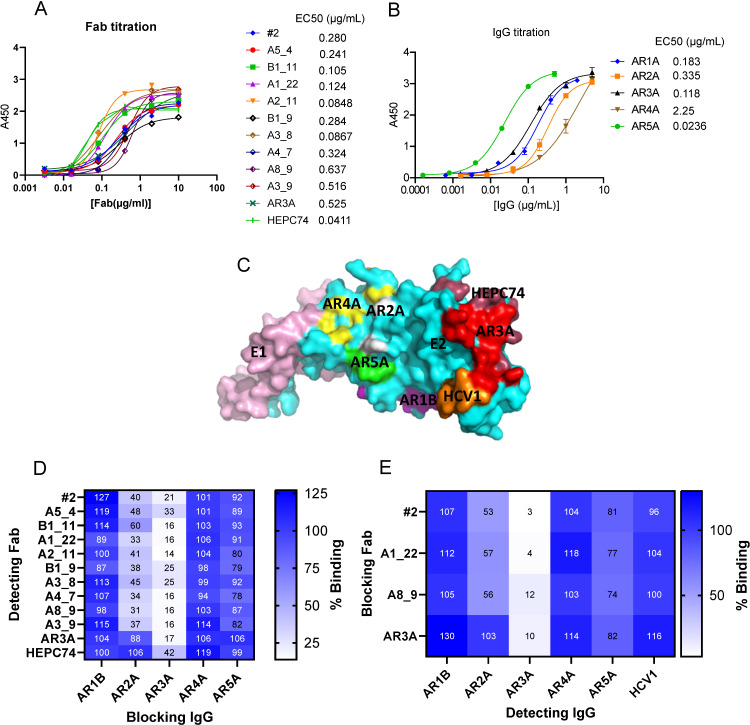
Epitope mapping of the Fabs. A) Titration of soluble Fabs to H77 E1/E2 with calculation of the half-maximal effective concentration (EC_50_) by four-parameter curve fitting using Graphpad Prism 10. B) Titration of blocking IgGs to H77 E1/E2 and calculation of EC_50_ by four-parameter curve fitting. C) Epitopes of the blocking IgGs as illustrated on the E1/E2 complex from PDB: 7T6X [31]. The epitope residues for AR1B, AR2A and AR5A are determined from alanine mutagenesis [32], while the AR4A epitope residues are determined from a cryo-EM map in complex with E1/E2 [31], the AR3A epitope is from x-ray crystallography in complex with the E2 core [12], the HEPC74 epitope overlaps partly with the AR3A epitope and is from x-ray crystallography in complex with the E2 ectodomain [33], and the HCV1 linear epitope (epitope I; residues 412-423) is from x-ray crystallography within the AS412 peptide [34]. D) Competitive ELISA by blocking with the reference full-length IgG at a saturating concentration and detecting the Fabs with streptactin-HRP at the EC_50_ in duplicate. The % binding value indicated is calculated as the percentage reduction of A450 signal measured from binding in competition with reference IgG compared to binding without addition of competitor. E) Reverse competition by blocking with the Fabs at saturating concentration and detecting the reference IgGs, as well as HCV1 at the determined EC_50_ with a HRP coupled Fc-specific antibody.

To express the antibodies in a human cell line, we subcloned the VH and VL of the Fabs into a dual promoter vector [29], modified to include a Twin-Strep-tag [30] at the C-terminus of the heavy chain constant region 1 (CH1). This modification facilitated both purification and detection of the Fabs in competition ELISAs against reference IgGs. Fab#17 and Fab A3_6 were excluded from further characterization as we were unable to express them in sufficient yields. Furthermore, B1_9 did not have an efficient pairing of the Hc and Lc, as there was a small fraction of unpaired Hc when run on a non-reducing SDS-PAGE (S1 Fig). We performed competition ELISA for the Fab panel in which we pre-incubated lectin-captured E1/E2 with either of the IgGs AR1B, AR2A, AR3A, AR4A, or AR5A, at a saturating concentration (Fig 3).

These previously characterized antibodies bind non-overlapping epitopes on E1/E2 [31,32]. Additionally, we included AR3A and HEPC74 in Fab format as references. To further validate the competition ELISA, we also reversed the assay by blocking with one Fab from each subgrouping and detecting with the reference IgGs, as well as HCV1, which targets epitope I, a linear epitope adjacent to AR3 (comprising residues 412-423) [34]. Notably, besides being blocked by AR3A, the panel was also blocked by AR2A IgG, which targets the back layer of E2 (Fig 3C).

**Fig 4:**
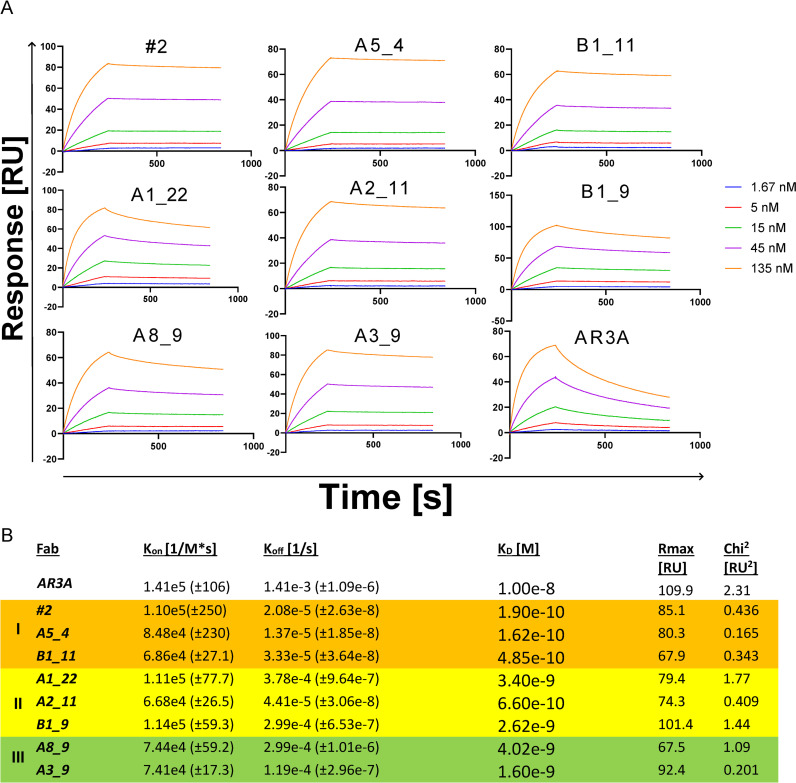
Binding kinetics of the Fab panel to sE2. Surface plasmon resonance (SPR) was used to determine the binding kinetics of the Fab panel to H77 sE2^384-645^ amine coupled to a CM5 chip on a Biacore T200. In A) the fitted SPR sensorgrams with the time from injection and the corresponding response. B) The calculated association (K_on_) and dissociation rate (K_off_) from the fitted sensorgrams. The grouping and coloring indicated are the same as determined in Fig 2. All isolated Fabs outcompete AR3A with the strongest affinity measured as 0.16 nM for A5_4 compared to 10 nM for AR3A.

As the AR2 and AR3 are found on the E2 glycoprotein, we investigated the binding kinetics of the panel of Fabs to membrane truncated soluble E2 (sE2: residues 384-645) of the H77 isolate. We purified monomeric sE2 via immobilized-metal affinity purification and size exclusion chromatography (S2 Fig). Using surface plasmon resonance (SPR), we measured the association (k_on_) and dissociation rate (k_off_) of the Fabs on a Biacore T200 system, calculating the dissociation constant (K_D_) (Fig 4).

The K_D_ of AR3A to sE2 was measured as 10 nM, which is similar to what has been reported by others [12]. The Fabs isolated from the patient library all exhibited higher affinity than the AR3A Fab, and Fabs in group I (#2, B1_11, A5_4) had K_D_ ranging from 0.16-0.49 nM. This exceptional affinity was primarily attributed to a very slow k_off_, with minimal Fab dissociation after 10 minutes.

### Neutralization potency and breadth of Fabs

**Fig 5:**
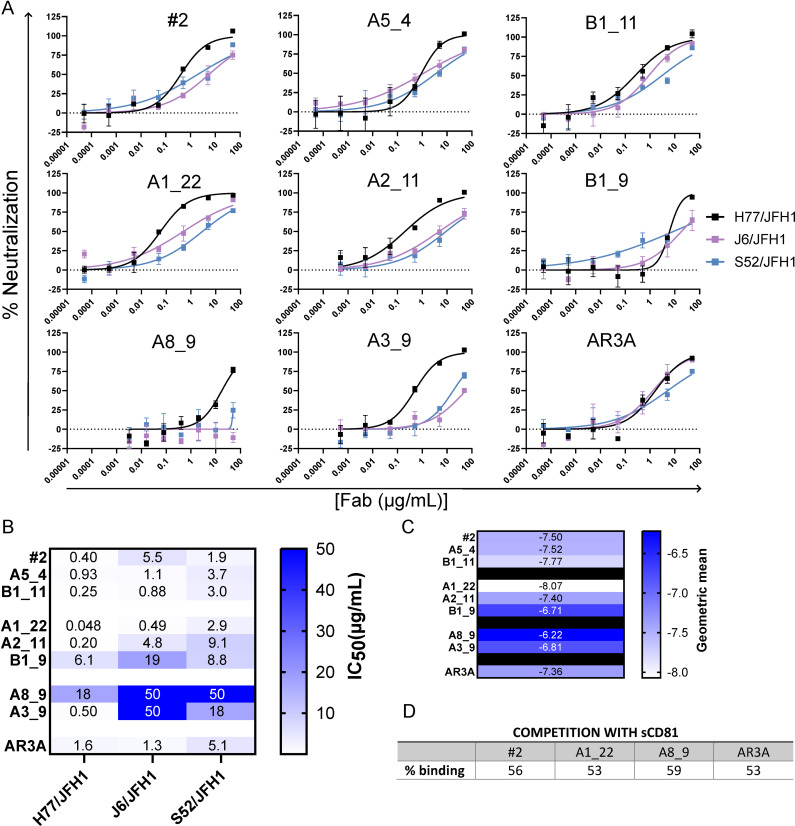
Fab neutralization of HCVcc isolates. A) The HCVcc recombinants H77/JFH1, J6/JFH1 and S52/JFH1 were subjected to a 5-fold dilution series of the Fabs, ranging from 50 μg/ml to 0.016 μg/ml, in triplicate. The data were analyzed using four-parameter curve fitting to obtain sigmoidal dose-response curves from which the half-maximal inhibitory concentrations (IC_50_) values were calculated. B) IC_50_ values for each Fab determined from the fitted curves. If IC_50_ could not be reliably determined, it was set to the maximum tested concentration of 50 µg/mL. C) The geometric mean calculated as the average of the molar logIC_50_ for the tested isolates. D) Competition ELISA to H77 E1/E2 with the large extracellular loop of CD81 fused to a human IgG_1_ Fc region (sCD81-Fc). With values of % binding determined as the A450 signal to H77 E1/E2 of Fabs blocked with sCD81-Fc divided by the signal of Fabs not blocked.

Intrigued by the superior binding kinetics of the novel AR3-like Fabs, we evaluated their neutralization potency in HCVcc assays against JFH1-based Core-NS2 genotype recombinants H77/JFH1 (Gt1a), J6/JFH1 (Gt2a), and S52/JFH1 (Gt3a), using AR3A Fab as reference (Fig 5).

All Fabs fully neutralized H77/JFH1 at the highest concentration tested (50 µg/mL), except for A8_9 (group III) for which a few HCV focus forming units (FFU) were observed even at the highest antibody concentration. Overall, neutralization efficacy against H77/JFH1 varied widely between the groups and the relative binding affinities measured against sE2 did not correlate with Fab neutralization potency. The Fabs in group I (#2, B1_11, A5_4) showed the strongest binding to H77 sE2, while they were slightly poorer neutralizers than A1_22 (group II) with the group I average IC_50_ to H77/JFH1 of 0.52 µg/mL compared to an average affinity to sE2 of 0.28 nM, corresponding to 0.014 µg/mL. Notably, A1_22 exhibited the highest neutralization potency against H77/JFH1, which in this instance did correlate well with its affinity to H77 sE2, K_D_ of 3.4 nM, corresponding to 0.17 µg/mL. These results illustrate that at these already high affinities, the relatively higher affinity to sE2 does not necessarily correlate with higher neutralization efficacy. Differences in neutralization potency were more pronounced when testing against heterologous strains. A1_22 demonstrated the best neutralization against J6/JFH1, while #2 was most effective against S52/JFH1. Notably, five Fabs outperformed AR3A in breadth of neutralization, with A1_22 exhibiting a 32-fold higher potency than AR3A against H77/JFH1. Finally, to elucidate the mechanism of neutralization we performed competition with the large extracellular loop of CD81 fused to a human IgG_1_ Fc region. CD81 is the main entry receptor for HCV and the CD81 binding site on E2 overlaps with the AR3A epitope [35]. We chose to test the best performing Fabs (#2 and A1_22) from group I and II as well as the worst performing Fab A8_9 from group III and AR3A as controls. All Fabs, including AR3A, competed equally well with the sCD81-Fc for binding to H77 E1/E2, indicating that the blocking of the receptor interaction is a likely mechanism for neutralization of the virus.

**Fig 6:**
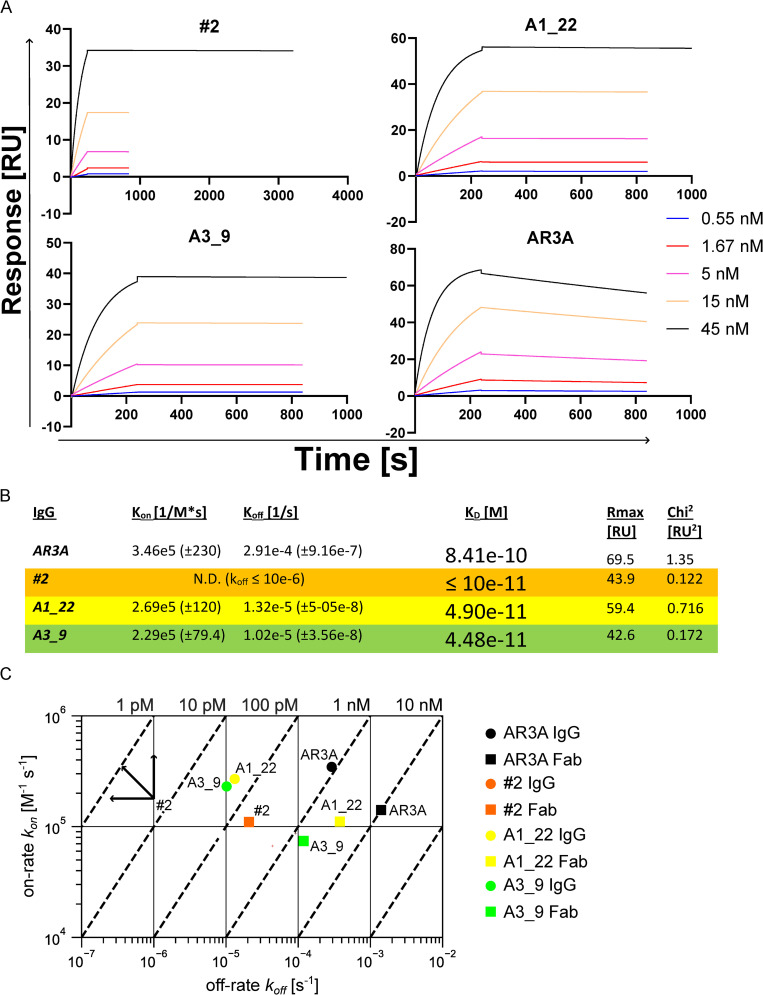
Kinetics of the IgGs to H77 sE2. A) Binding kinetics to H77 sE2^384-645^ was determined using a CM5 chip on a Biacore T200. A) The fitted SPR sensorgrams with the time from injection and the corresponding response. IgGs injected at 45 nM highest concentration; 240s at 60 μL/min with 600s or 3600s dissociation times. B) The calculated association (K_on_) and dissociation rate (K_off_) from the fitted sensorgrams. C) On-off rate map displaying the association (*k*_*on*_) and dissociation (*k*_*off*_) rates for a subset of IgG and Fabs measured at 25 °C. Each color represents a different Fab, IgG pair with Fabs shown as squares and IgGs as circles. The dotted lines on the diagonals represent different dissociation constants (*K*_*D*_, where *K*_*D*_ = *k*_*off*_*/k*_*on*_) with representative values shown at the top of the graph. For the #2 with dissociation constants below the limit of detection of the instrument, the arrows point to the regions of the map where values would be located.

### Binding kinetics, neutralization potency and breadth as full-length IgG_1_

The best performing Fab from each grouping in terms of affinity and neutralization potency were converted to full-length IgG_1_ for further characterization (#2, A1_22 and A3_9). First, we tested the kinetics of the IgGs and compared to the Fab format (Fig 6).

**Fig 7:**
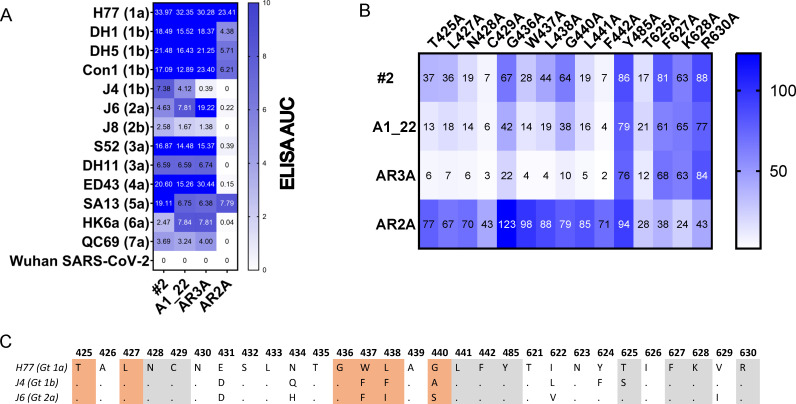
Binding specificity of the isolated IgGs. A) Titration of IgGs to detergent-extracted E1/E2 from different isolates and the spike protein from Wuhan SARS-CoV-2 as a negative control in a 3-fold dilution series from 10 µg/mL to 0.04 µg/mL. The area under the curve values displayed are calculated using Graphpad Prism 10. B) Alanine-scan mutagenesis of residues reported to be affecting binding of either AR2A or AR3A by Pfaff-Kilgore et al. [36]. The binding is shown relative to A450 signal in ELISA to wildtype H77 at the EC_50_ of the tested IgGs and normalized using the signal of HCV1 at EC_50_. C) Sequence alignment of the E2 residues surrounding the tested alanine-scan constructs of H77, J4 and J6. The residues tested by alanine-scan of H77 E1/E2 are marked in grey while the residues that differed in binding dependence between A1_22 or #2 and AR3A are marked in orange.

For all Fab binders, including AR3A, affinity was increased at least 10-fold in the full-length IgG format. For #2, we could not measure the kinetics precisely, as the K_*off*_ reached the limit of detection for the Biacore T200 (Detection limit of 10^-5^ to 1 s^-1^ for k_*off*_). Additionally, the off rate for #2 was so slow that there barely was decay on the response even after 1 hour. We estimated the *K*_*D*_ of #2 to H77 sE2 close to 1 pM, and A1_22 and A3_9 around 45-49 pM, which all were 10 to 100-fold stronger than the *K*_*D*_ of AR3A. The A3_9 IgG did not express to a high yield, and we continued the characterization only with #2 and A1_22, in which they were further purified by size exclusion chromatography (S3 Fig). We tested the cross reactivity of AR2A, AR3A, #2 and A1_22 by titrating the IgGs to detergent-extracted E1/E2 from a panel of isolates in ELISA and used the spike protein from the Wuhan SARS-CoV-2 as a negative control (Fig 7).

A1_22 and #2 were reactive to all tested E1/E2 constructs. AR3A reacted to all tested constructs except for J4, where only limited signal was observed at the highest concentration. A1_22 and #2 were reactive to J6, although the binding was reduced compared to AR3A. We further tested the contact sites on E1/E2 by performing an alanine scan mutagenesis for the residues involved with binding of either AR2A or AR3A [36]. Overall, the residues on E1/E2 critical for binding are similar for the IgGs isolated here and AR3A, although with significant differences for T425, L427, G436, W437, L438 and G440. Specifically, the residues G436, W437 and L438 are also not conserved across the H77, J4 and J6 isolates, which could explain the differences in reactivity seen across these isolates. Although the IgGs seemingly do not engage with the same residues as AR2A, we do note that most residues in the E1/E2 complex cannot be effectively mutated without compromising the overall fold of the protein [36]. We speculate that the IgGs engage the front layer of E2 in an angle that at least occludes the AR2A epitope and might interact with residues surrounding this epitope.

**Fig 8:**
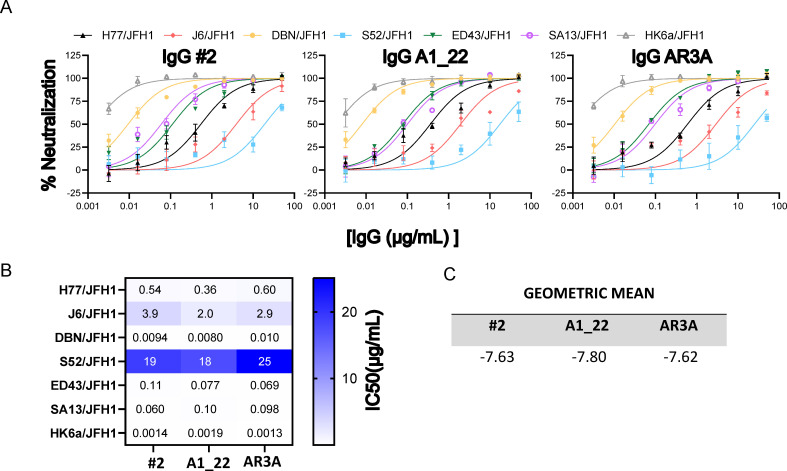
IgG neutralization of HCVcc isolates. A) The HCVcc recombinants H77/JFH1, J6/JFH1, DBN/JFH1, S52/JFH1, ED43/JFH1, SA13/JFH1 and HK6a/JFH1 were subjected to a 5-fold dilution series of the IgGs, ranging from 50 μg/ml to 0.016 μg/ml, in triplicate. The data were analyzed using four-parameter curve fitting to obtain sigmoidal dose-response curves from which the half-maximal inhibitory concentrations (IC_50_) values were calculated. B) IC_50_ values for each IgG determined from the dose-response curves. C) The geometric mean calculated as the average of the molar logIC_50_ (lower values correspond to more potent neutralization) for the tested HCVcc recombinants.

Finally, to benchmark the antibodies against AR3A, we performed an extensive evaluation of their neutralization efficacy against HCV genotypes of the clinically relevant Gts 1-6 using cell culture infection systems (Fig 8).

The panel of HCVcc was chosen based on genetic variability and also covers antigenic variability by including the generally most neutralization resistant variants (J6 and S52), as well as the most neutralization sensitive variants (SA13 and HK6a) [6]. As we observed an increase in affinity across all antibodies from Fab format to the full-length IgG format, we expected to see a similar trend for neutralization potency. However, for #2 and A1_22 the potency was similar in the IgG format and even slightly decreased against S52, whereas AR3A was slightly more potent in the IgG format. Overall, the antibodies performed remarkably similar to AR3A across all tested genotypes in the IgG format.

## Discussion

In this study, we isolated a panel of novel AR3-like bNAbs from the same V_H_1-69 germline using a phage display Fab library from pt. H. These antibodies all compete for binding with AR3A and AR2A antibodies and several have neutralization potencies that compares to that of the canonical and potent AR3A antibody. Our findings add to the growing body of literature on HCV-specific antibodies and shed light on the potential importance and large diversity of AR3-like bNAbs in HCV vaccine development.

Another phage display library derived from pt. H had previously been used to isolate five unique Fabs by panning against sE2 [27]. Three of these Fabs, HCV#4, HCV#7, and HCV#12 were derived from the V_H_1-69 germline (also included in Fig 2C and S1 Data) as was the case for all antibodies identified in this study. HCV#4 was similar to group II Fabs identified here, whereas groups I and III had not been observed before, showing that the patient antibody repertoire had not been exhaustively panned for potentially cross-genotype reactive bNAbs. HCV#4 and HCV#7 blocked the entry receptor CD81 from binding to sE2, while HCV#12 did not. Therefore HCV#4 and HCV#7 were tested for neutralization. At the time, HCVcc was not yet fully established and these Fabs were tested against HCV pseudo particles from Gt1a, Gt1b, Gt2a, Gt3a, Gt4a, and Gt5a. However, the Fabs isolated previously were poor neutralizers as they were only able to neutralize Gt1a and Gt1b, while HCV#4 also neutralized Gt2a. In this study, we used lectin immobilized full-length E1/E2 directly from the detergent-extracted cell lysate to retrieve additional monoclonal antibodies from pt. H. While we panned against E1/E2 from both homologous H77 and the heterologous S52 isolate, this did not result in observable differences in the sequences of selected clonal binders, indicating that while the H77 strain was isolated from the same patient, the antibody repertoire had changed significantly in the time between isolation of the H77 viral sequence and the construction of the library. It is worth noting that the method of lectin-based immobilization of E1/E2 glycoproteins used to perform the pannings could have influenced the selection of binders by orienting the glycoprotein complex in a way that favors the binding of AR2 or AR3 targeting binders. Specifically, most of the glycans are situated on the opposite side of these epitopes depicted in Fig 3C [37]. Additionally, the exceptionally high affinity of the isolated binders to sE2 could have outcompeted other antibodies present in the constructed library from pt. H. As such, using alternative antigens or methods for presentation of the antigen could possibly lead to isolation of a more diverse set of binders.

Many screening efforts for HCV antibodies have previously been performed. Law et al. [11] isolated a panel of antibodies recognizing three non-overlapping regions, AR1-AR3, by panning an immune phage display library against sE2. Subsequently, Giang et al. [10] isolated antibodies from the same phage display library recognizing AR4 and AR5 by panning against full-length E1/E2 and continuously masking the epitopes of binders recognizing AR1, AR2, and AR3. In parallel, Keck et al. [38] used surface yeast display to isolate a panel of antibodies recognizing the antigenic domains A-C, which do not correspond exactly to the ARs but where domain A overlaps with AR1, domain B overlaps with AR3, and domain C overlaps with AR2. The same library was then used against E2 variants with substitutions that abolished binding of domain A and B antibodies, to select the domain D antibodies, which is an epitope within the CD81 binding site of E2 adjacent or slightly overlapping with AR3/domain B epitopes [39]. These studies illustrate the high immunogenicity of domain B/AR3 epitopes and the need for sophisticated screening strategies when interrogating the B-cell repertoire of patients infected with HCV. This study further confirms these findings, as we only retrieved domain B/AR3-like antibodies when panning directly to either homologous or heterologous E1/E2. Intriguingly, the entire panel of novel E1/E2 binders could be blocked by both AR3A and AR2A. Recently, Ogega et al. [40] isolated several anti-E2 bNAbs from patients that had cleared acute infection. Interestingly, they found several back-layer targeting bNAbs, where specifically a V_H_4-34 bNAb, hcab40, targeted both the front layer and back layer of E2 in the crystal structure. The paratope of Hcab40 completely blocks the putative epitope of AR2A, while also making contact with front layer residues H445 and K446, although not blocking AR3C. Additionally, Weber et al. [41] isolated V_H_1-69-derived AR3-like bNAbs from chronically infected donors, showing that most bNAbs utilize this germline family. The two most broad and potent antibodies, mAb1198 and mAb1382 (also included in S1 Data), interacted with both the front layer and the back layer of E2. In this study, we could only pinpoint residues in the E2 front layer involved with interactions of the isolated antibodies and none of the tested back layer residues led to a loss in binding by alanine-scan. This is complicated by the fact that most residues on E2 and especially in the back layer are critical for the correct overall fold of E2 [36]. The back layer of E2 constitutes the residues C597-C644 and by mutating 27 of these 46 residues to alanine, the binding of AR3A is reduced by more than 50% although the AR3A antibody does not interact with any of these residues. In our study, we show that the antibodies compete for binding with both AR3A and AR2A, which can be interpreted in two ways. First, the antibodies could bind to the AR3 epitope at an angle that leads to indirect partial steric occlusion of the AR2 epitope. Secondly, the antibodies could bind to both the AR3 epitope and the AR2 epitope. Without structural information, we are unable to distinguish between these two possibilities. However considering the exceptional affinity of the Fabs described here to sE2, it is likely that these antibodies interact with a larger E2 surface area, which lends support to the interpretation that additional non-AR3 residues could be involved in binding. The differences in the paratope between these new Fabs and AR3A is further supported by the cross-reactivity testing in which the isolated antibodies recognized all tested isolates, whereas AR3A was reactive with all isolates, except J4, a genotype 1b isolate, which has a G440A mutation in the E2 protein. When introducing the G440A mutation in H77 this abolished binding to E1/E2 for AR3A, but only led to reduced binding for A1_22 and #2, further supporting epitope differences between this class of antibodies and AR3A. Additionally, only the mutation of C429 and F442 completely abolished binding of A1_22 and #2, whereas the remaining mutations in the front layer residues only led to reduced binding, which further indicates epitope differences compared to AR3A.

Interestingly, in the study by Weber et al. [41], they highlight the critical role of somatic mutations within the HCDRs, particularly that mutations in HCDR1, at positions S30 and S31, were involved with increased neutralization potency. This fits well with the antibodies in this study, as group I antibodies carried the mutations S30T and S31L, group II S30N and S31I, while group III, also being the worst performing in neutralizations, still carried the germline encoded S30 for some of the group members. Contrary to the study by Weber et al. [41], F54 mutations in the HCDR2 seemed to affect neutralization potency negatively, while in our study all the isolated antibodies carried a F54V mutation. The results of these prior studies taken together with the results reported here, further emphasizes the potential significance of inducing back layer targeting bNAbs in an HCV vaccine.

By investigating binding kinetics to H77 sE2, the Fabs revealed exceptionally high affinities, surpassing the reference AR3A Fab, with some of the Fabs in the panel displaying a K_D_ in the sub-nanomolar range. This was increased even further when the antibodies were converted to IgG, as the affinity to H77 sE2 in this format surpassed the lower limit of detection for the instrument. This was mainly driven by an exceptionally slow off-rate with almost no decay even after an hour. The high affinity measured to sE2 did not correlate to neutralization potency against HCVcc. Specifically, it is interesting that Fabs in group II (A1_22, A2_11, and B1_9), which have the same exact Hc but distinct Lc, are blocked by AR2A and AR3A to the same degree, and have the same affinity to sE2, but none-the-less have very different neutralization profiles. For B1_9 this reduced neutralization potency could be linked to poor pairing efficiency of the Hc/Lc (the Hc and Lc paired well in all other tested Fabs) (S1 Fig). The Hc/Lc pairings of the isolated Fabs are a result of the random reassortment during library construction, which also means that it is unclear whether the observed Hc/Lc pairings were found in the patient. Most of the V_H_1-69 bNAbs isolated against HCV, Influenza, or HIV-1 interact primarily through Hc interactions and are often independent of the Lc [19]. However, other recent studies have shown that this is not always the case. Tzarum et al. [42] showed that the binding to E2 was abolished by replacing the Lc of HC11, 212.1.1, HC1AM, HC84.26AM and HC84.1, while AR3A and 212.10 were completely independent of the Lc. Similarly, Capella-Pujol et al. [43] showed that AR3C binding and neutralization was independent of Lc pairing, while HEPC74 binding and neutralization was significantly reduced upon swapping the Lc. In this study, the group II Fabs in the panel may be dependent on the Lc, which could be due to direct interaction of the Lc with E2 or Lc induced conformational change of the Hc [44,45].

In summary, we identified a novel class of AR3-like HCV antibodies that block the AR2A epitope and demonstrate higher neutralizing potency than the reference AR3A Fab, as well as an exceptional affinity to HCV E2 as Fabs and, in particular, as full-length IgGs. The observed potency and affinity of these antibodies further reinforces the importance of inducing AR3-like antibodies in an HCV vaccine, due to their broad and potent neutralization of HCV, as well as accentuating the complexity of this class of antiviral antibodies. The findings of this study are of high relevance to consider in the rational design of a protective HCV vaccine.

## Methods

### Ethics statement

The patient was enrolled in an IRB NIAID internally approved protocol. The patient signed informed consent. Such consent can only be obtained in IRB approved studies. The protocol was submitted under 99-CC-0090, entitled: Generation of Anti-HCV antibodies from Bone Marrow Defining the Repertoire of Immune Response to HCV Quasispecies.

### Human Fab antibody library construction

Lymphocytes from bone marrow, aspirated from a chronic HCV patient, patient H (pt. H), were isolated on a Ficoll gradient. Total RNA was extracted from 1X10^7^ lymphocytes using RNeasy mini kit (Qiagen, Cat. No.: 74104). The 1st strand cDNA was reverse transcribed with oligo(dT) primer using a First-Strand cDNA Synthesis Kit (Cytiva, Cat. No.: 27926101) and used as a template for antibody Fab coding fragment amplification. Briefly, the γ1 heavy chain Fd (variable and first constant region) was amplified with nine human heavy chain–specific 5´ primers and a human γ1–specific 3´ primer. The κ chain was amplified by PCR with seven human κ chain–specific 5´ primers and a 3´ primer matching the end of the constant region [46]. The λ chain was amplified by PCR with six human λ chain–specific 5´ primers and a 3´ primer matching the end of the constant region [47]. PCR was performed for 30 cycles of 95°C for 1 min, 52°C for 1 min, and 72°C for 1 min with HotStart Taq DNA polymerase (Qiagen, Cat. No.: 203203). Amplified κ and λ chain DNA fragments were pooled, purified, digested with *SacI* and *XbaI*. The restriction enzyme digested light chain fragments were then ligated into the pComb3H vector with T4 DNA ligase (NEB, Cat. No.: M0202L) [48]. The recombinant plasmid DNA was introduced into *E. coli* Top 10 cells (Lucigen) by electroporation, yielding 1 X 10^7^ individual clones. The plasmid DNA containing light chain sequences was digested with *XhoI* and *SpeI*, ligated with γ1 Fd DNA cut with the same enzymes. The plasmid DNAs containing light and heavy chains were transformed into *E. coli* Top 10 cells by electroporation. Thus, a phage display Fab library with a size of 2.4 X 10^8^ individual clones was generated.

### Phage display bio-panning

E1/E2 antigens or an empty vector were transiently transfected into HEK293T cells and following 48 hours, membrane proteins were extracted with 1X NativePAGE sample buffer (Thermo Scientific, Cat.No.: BN2003) and 1% n-Dodecyl-β-D-Maltoside (Thermo Scientific Cat.No.: 89902). 5 µg/mL *Galanthus nivalis* lectin (Sigma-Aldrich, Cat.No.: L8275) was coated on MaxiSorp (Thermo Scientific, Cat.No.: 442404) plates overnight and used to immobilize the antigens. The phage display library was panned by depleting the library using lectin-immobilized cell lysate (empty vector) before transferring to lectin-immobilized E1/E2. Washing was performed with increased stringency for each round (i.e., 10 times with PBS first round, 20 times with PBS second round and 30 times with PBS the following rounds). The bound phages were eluted with 10 mM glycine-HCl, pH 2.7 (Invitrogen, Cat.No.: 15527013) and neutralized with 2M Tris-HCl pH 8 (Invitrogen, Cat.No.: 15506017) before infecting log-phase TG1 cells. The infected TG1 cells were either plated on LB+100 µg/ml ampicillin plates for selection of individual clones or superinfected with M13KO7 and concentrated with PEG6000+2.5M NaCl for subsequent panning rounds. Monoclonal ELISA was performed by growing single colonies in 96-well deep wells at 37°C overnight, then diluted 1:100 in 2xYT supplemented with 100 µg/mL ampicillin and 1% glucose and grown to log-phase before being superinfected with M13KO7 and grown at 30°C overnight in 2xYT supplemented with 100 µg/mL ampicillin and 50 µg/mL kanamycin. The remaining overnight culture was stored in 25% glycerol at -80°C. The superinfected clones were spun down, and the supernatant was used in ELISA. The ELISA was performed by immobilizing E1/E2 or cell lysate on lectin-coated Maxisorp plates and detected using anti-M13 HRP (Sino Biological). E1/E2-positive clones were then grown from glycerol stocks and phagemid DNA was extracted with mini prep spin-column kit (Qiagen).

### Expression and purification of Fabs

The VH and VL from the isolated phagemids were cloned into a dual promoter vector [29] that we had modified to contain the human CK and CH1 with a C-terminal Twin-Strep tag [30] for Fab production. The sequence for AR3A was retrieved from Genbank: 1545825865 and HEPC74 from Genbank: 1519485883 and the gene product was constructed by overlap-extension PCR and subcloned into the modified pVitro vector for parallel production and experimentation. The soluble Fabs were then produced by transfecting HEK293F cells for 72 hours before collecting the supernatant and running it over a Streptactin-XT flow column attached to the Äkta Pure system according to manufacturer’s instructions. The eluted Fab fraction was subsequently concentrated in an Amicon ultracentrifugal filter with a 30 kDa cutoff and desalted using Zeba desalting spin columns. The concentration was determined by measuring the absorbance at 280 nm on a Nanodrop and using values for the extinction coefficients computed from the corresponding amino acid sequences by the ProtParam program http://web.expasy.org/protparam.

### Expression and purification of IgGs

The VH and VL were cloned in frame into the rest of the IgG1 or kappa constant regions in a dual promoter vector [29] and transfected into HEK293F cells for 3 days before collecting the supernatant and running it over a protein G column attached to the Äkta Pure system. The bound IgGs were washed with PBS and eluted with 10mM glycine-HCl, pH 2.7 into 1M Tris-HCl, pH 8. The IgG containing fraction was concentrated in an Amicon ultracentrifugal filter with a 100 kDa cutoff and run into PBS over the Superdex 200 10/300 column attached to the Äkta Pure system. The IgG containing fractions were analyzed by Coomassie-staining a non-reducing SDS PAGE gel and the monomeric species were pooled and concentrated in an Amicon ultracentrifugal filter with a 100 kDa cutoff. The purity was verified in a coomassie stained non-reducing SDS PAGE gel and the concentration was determined in a similar way as for the Fabs.

### Expression and purification of sE2

The transmembrane-truncated soluble E2 (sE2, residues 384-645) from H77 [25], was cloned into a phCMV vector downstream of a tPA signal peptide sequence and with a C-terminal GSSGHHHHHH tag. The construct was transfected into HEK293F cells. Three days later the supernatant was collected and diluted 1:1 with wash buffer (50 mM sodium phosphate pH 7.4, 300 mM sodium chloride, 20 mM imidazole). This was run over a HisTrap HP column attached to the Äkta Pure system, washed with the wash buffer for 10 column volumes and eluted with a gradient elution over 20 column volumes with elution buffer (50 mM sodium phosphate pH 7.4, 300 mM sodium chloride, 500 mM imidazole). The sE2 containing fractions were pooled and concentrated using Amicon ultracentrifugal filters with a 30 kDa cutoff and run into PBS over the Superdex 200 10/300 column attached to the Äkta Pure system. We analyzed the fractions by non-reducing Coomassie-stained SDS PAGE gel and pooled all the monomeric sE2 fractions and concentrated these using Amicon ultracentrifugal filters with a 30 kDa cutoff. We verified the purity by denaturing sE2 at 90°C for 10 min with 10mM DTT, we then ran it alongside a non-reduced sE2 on an SDS-PAGE detected by Coomassie-staining. The concentration was determined in a similar way as for the Fabs.

### Competition ELISA

Titration curves for the Fabs and IgGs were determined against H77 E1/E2 and the EC_50_ determined by fitting a four-parameter logistic curve in Graphpad Prism 10. The reference IgGs (AR1B, AR2A, AR3A, AR4A, or AR5A) were provided by Mansun Law (Scripps Research Institute, San Diego, USA [10,11]). Lectin-coated plates were blocked with 1% BSA and 0.2% skim-milk powder (BSK) and incubated with H77 E1/E2 containing cell lysate for 1 hour, then the plates were washed and incubated for 1 hour with the blocking IgG or Fab at a saturating concentration. Finally, the Fabs or IgGs were incubated at EC_50_ for 1 hour, then the plates were washed, and detected with either Streptactin-HRP (IBA, Cat. No.: 2-1502-001) or Peroxidase AffiniPure Goat Anti-Human IgG, Fcγ fragment specific (Jackson ImmunoResearch, Cat. No.: 109-035-098). The residual binding was calculated as signal from Fab blocked by the reference IgG divided by Fab binding to H77 E1/E2 multiplied by 100. The recombinant human CD81 LEL Fc chimera protein (R&D systems, Cat. No.: 9144-CD) was used in competition ELISA with the Fabs at a saturating concentration.

### Alanine scan-mutagenesis

The H77 E1/E2 residues were mutated by inverse PCR containing mutations to alanine at the 5´ end and ligated with KLD enzyme mix (NEB, Cat.No.: M0554S). The mutated constructs were transformed into top10 *E. coli* and verified by sequencing before being transfected into HEK293-T cells and extracted with 1X NativePAGE sample buffer and 1% n-Dodecyl-β-D-Maltoside for lectin capture ELISA. The EC_50_ value of IgGs to H77 E1/E2 was used to incubate with the mutated constructs and the signal was normalized to the signal of HCV1 at EC_50_ to account for differences in expression level.

### Cell culture virus antibody neutralization

The Huh7.5 cells [49] were provided by Charles Rice (The Rockefeller University, New York, USA). The cells were cultured in Dulbecco’s modified Eagle’s medium (DMEM) (Gibco/Invitrogen) supplemented with 10% heat-inactivated and filtered fetal bovine serum, 100 µg/mL streptomycin and 100 U/mL penicillin (Gibco/Invitrogen) grown at 37°C with 5% CO_2_ and split every second to third day. To test the neutralization potential of the Fabs, we used HCVcc JFH1-based Core-NS2 recombinants of H77/JFH1 [25], J6/JFH1 [50], and S52/JFH1 [26]. For the full-length IgGs, we expanded the panel to also include DBN1/JFH1 [51], ED43/JFH1 [25], SA13/JFH1 [52], and HK6a/JFH1 [22]. For each HCVcc recombinant, we generated first passage envelope protein sequence-confirmed virus stocks, as described by Olesen et al. [53]. Briefly, 2 × 10^5^ Huh7.5 cells were plated in a 6-well format and incubated at 37˚C at 5% CO_2_ for 24 hours. The cells were infected with 500 µl virus containing supernatant collected 4 days post transfection. At the peak of infection, supernatants from two time-points were collected from which the envelope protein sequences were determined with Sanger sequencing (Macrogen Europe) and the infectivity titer was determined.

The neutralization assays were performed as described by Olesen et al. [53]. Briefly, we plated 7 × 10^3^ Huh7.5 cells in a 96-well format. The next day, we prepared a 5-fold dilution series of the antibodies in either Fab or IgG1 format starting at 50 µg/ml. Antibodies were mixed with the virus stocks (readout 50-200 FFU/well) and incubated for 1 hour at 37˚C and 5% CO_2_. Next, the antibody/virus mix, and 8 wells of virus only were added to the cells for 4 hour infections. Following incubation, the cells were washed with PBS and new media was added for a final incubation of 48 hours. The cells were fixed and stained for HCV NS5A with 9E10 antibody (Cell Essentials). HCV FFUs were counted and normalized to virus only and analysed with sigmoidal dose-response (variable slope) curves with bottom constraints set to 0 and top constraints set to 100 using GraphPad Prism 10. The IC_50_ values were calculated from the fitted curves and the standard error of the logIC_50_ was calculated by a symmetrical 95% confidence interval. To compare the fitted curves across IgGs, we performed an ordinary one-way ANOVA (Tukey test) using the logIC_50_, with the standard error and degrees of freedom calculated from the fitted curves.

### Surface plasmon resonance (SPR)

All SPR experiments were performed at 25 °C on a Biacore T200 instrument equipped with CM5 sensor chips (Cytiva, Uppsala, Sweden). SPR running buffers and amine-coupling reagents (N-ethyl-N’-(3-dimethlyaminoproply)carbodiimide (EDC), N-hydroxysuccinimide (NHS), and ethanolamine HCl) were purchased from Cytiva. 1x PBS-P (11.9 mM NaH2PO4-Na2HPO4 pH 7.4, 137 mM NaCl, 2.7 mM KCl, 0.005% (v/v) surfactant P20) was used as running buffer for all experiments. H77 sE2^(384-645)^ at 10 μg/mL in 10 mM sodium acetate pH 5.5 was amine coupled on the flow cell 2 (Fc2) channel of a CM5 sensor chip at ~ 300 RU or for Fab binding experiments. The flow cell 1 (Fc1) remained unmodified and was used as reference for subtraction of systematic instrumental drift. Fabs threefold serial dilutions (from 135 to 1.65 nM) were prepared in the SPR running buffer from the primary stocks. The Fabs were injected sequentially over the two flow cells at a flow rate of 60 μl/min for 240 s. The dissociation rate of the complexes was monitored for 600 s. For slow dissociation rate Fab complexes (#2, A5_4, B1_11, and A2_11), three injections of the Fabs at the highest 135 nM concentration tested were injected at the end of the concentration series experiment and the dissociation rate of the complexes was monitored for 1 h. Data processing and fitting was done using the BiaEvaluation software (v. 3.2.1, Cytiva, Uppsala, Sweden). The raw sensorgrams were double referenced (referring to the subtraction of the data over the reference surface and the average of the buffer injections from the binding responses). The association and dissociation phases were globally fitted using a 1:1 interaction model yielding single values for the ka and the kd. The equilibrium dissociation constant, KD, is the rate of kd over ka. #2, A1_22, and A3_9 IgGs threefold serial dilutions (from 45 to 0.55 nM) were prepared in the SPR running buffer from the primary stocks. The Fabs and all IgGs were injected sequentially over the two flow cells at a flow rate of 60 μl/min for 240 s. The dissociation rate of the complexes was monitored for 600 s. For the slow dissociation rate Fab complexes (#2, A5_4, and B1_11, and A2_11) and for the four IgGs tested, three injections at the highest (135 nM for Fabs or 45 nM for IgGs) concentration tested were injected at the end of the concentration series experiment and the dissociation rate of the complexes was monitored for 1 h. One single 18 s injection at 10 μL/min of 10 mM glycine pH 2.0 regeneration solution strips the remaining analyte (Fabs or IgGs) at the end of each binding cycle.

## Supporting information

S1 Data Chothia numbered VH sequences of isolated antibodies and published VH1-69 derived bNAbs. (XLSX)

S1 Fig Fab light-chain pairing. The Fabs were expressed in HEK293F cells, and the supernatant was run under either non-reducing conditions or with 25mM DTT followed by heating at 90°C for 10 minutes. Western blot signal was detected with streptactin-HRP and developed with either pico chemiluminescent substrate for 2 seconds for the group I and II Fabs or with atto chemiluminescent substrate for 300 seconds for the group III Fabs. The bands containing light chain paired Fd (VH1-CH1) or unpaired Fd are indicated by arrows. Only B1_9 contained unpaired Fd under non-reducing conditions.
(PNG)

S2 Fig sE2 purification. sE2 SEC chromatogram of immobilized metal affinity purified H77 sE2 (residues 384-645) and Coomassie-stained SDS PAGE of denatured and reduced with 10mM DTT (+) and non-reduced (-) sE2.
(TIFF)

S3 Fig IgG purification. Full-length IgG SEC chromatogram and Coomassie-stained non-reducing SDS PAGE.
(TIFF)
