# Supplementary figures and images for: Selection and characterization of a broadly neutralizing class of HCV anti-E2 VH1-69 antibodies
Source: PLoS Pathog. 2025 Mar 28;21(3):e1012428. doi: 10.1371/journal.ppat.1012428 (PMC11999149; doi:10.1371/journal.ppat.1012428)

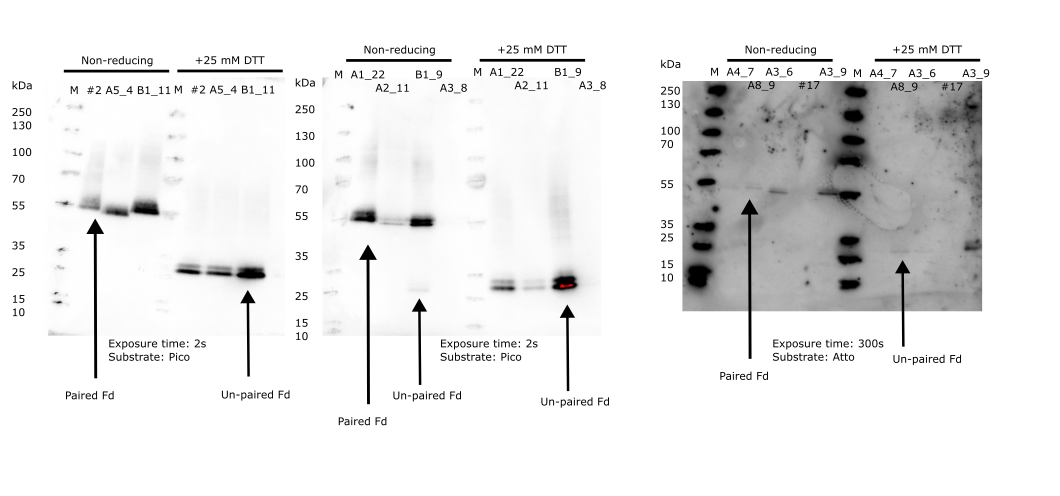

Supplement: S1 Fig — The Fabs were expressed in HEK293F cells, and the supernatant was run under either non-reducing conditions or with 25mM DTT followed by heating at 90°C for 10 minutes. Western blot signal was detected with streptactin-HRP and developed with either pico chemiluminescent substrate for 2 seconds for the group I and II Fabs or with atto chemiluminescent substrate for 300 seconds for the group III Fabs. The bands containing light chain paired Fd (VH1-CH1) or unpaired Fd are indicated by arrows. Only B1_9 contained unpaired Fd under non-reducing conditions. (PNG) [file ppat.1012428.s002.png]

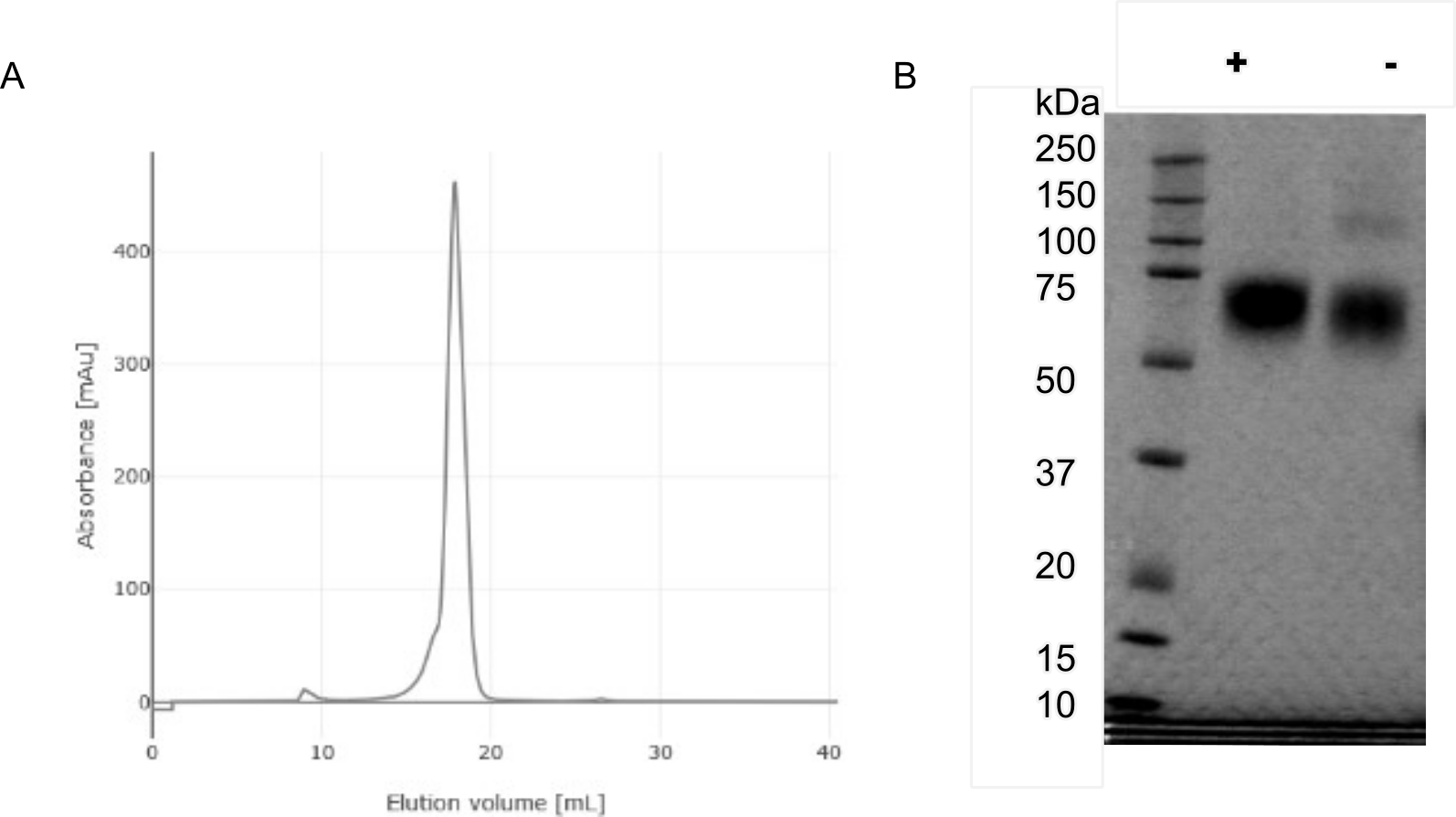

Supplement: S2 Fig — sE2 SEC chromatogram of immobilized metal affinity purified H77 sE2 (residues 384-645) and Coomassie-stained SDS PAGE of denatured and reduced with 10mM DTT (+) and non-reduced (-) sE2. (TIFF) [file ppat.1012428.s003.tiff]

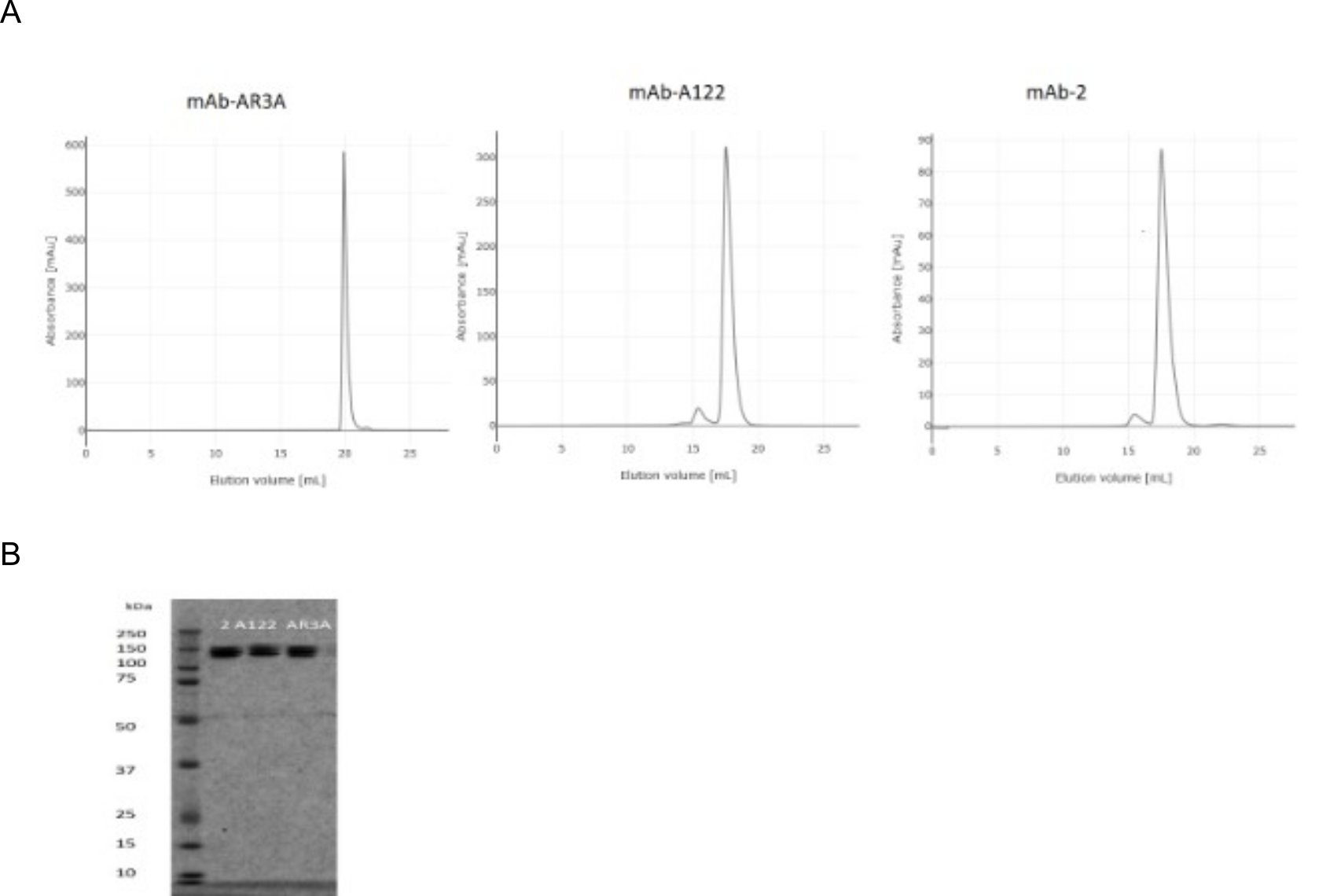

Supplement: S3 Fig — Full-length IgG SEC chromatogram and Coomassie-stained non-reducing SDS PAGE. (TIFF) [file ppat.1012428.s004.tiff]
